# Association between Homocysteine Levels and All-cause Mortality: A Dose-Response Meta-Analysis of Prospective Studies

**DOI:** 10.1038/s41598-017-05205-3

**Published:** 2017-07-06

**Authors:** Rui Fan, Aiping Zhang, Fade Zhong

**Affiliations:** 1 Department of medical quality, Ningbo Medical Center Lihuili Eastern Hospital, Ningbo, Zhejiang, 315042 China; 2 Department of chronic disease control and prevention, Zhenhai Center for Disease Control and Prevention, Ningbo, Zhejiang, 315200 China; 3 The Central Blood Station of Ningbo, Ningbo, Zhejiang, 315099 China

## Abstract

Plasma homocysteine (Hcy) levels may be associated with all-cause mortality risk. However, the results of this association are conflicting and the dose-response relationship between them has not been clearly defined. In this meta-analysis, we conducted a systematic literature search of the PubMed, Embase, Web of Science and Cochrane Library for the relevant articles dated up to February 2017. Pooled relative risks (RRs) and corresponding 95% confidence intervals (CIs) were calculated to evaluate the estimates, and the dose-response relationship was estimated using a restricted cubic spline model. Eleven prospective studies (4,110 deaths among 27,737 individuals) were included. The summary RR of all-cause mortality for the highest Hcy category vs. the lowest Hcy category was 1.80 (95% CI: 1.51, 2.14) with the random effects model. In dose-response meta-analysis, Hcy levels were significantly associated with all-cause mortality risk in a linear fashion (*p*_nonlinearity_ = 0.255), and the risk of all-cause mortality increased by 33.6% for each 5 µmol/L increase in Hcy levels (RR = 1.336, 95% CI: 1.254–1.422, *p* < 0.001). Findings from this dose-response meta-analysis suggest that Hcy levels are linearly and positively associated with risk of all-cause mortality.

## Introduction

Homocysteine (Hcy), a sulphur-containing amino acid, is biosynthesized from methionine via a multi-step process^1^. A high level of homocysteine in the blood (hyperhomocysteinaemia) is correlated with a wide range of diseases, including cardiovascular disease, renal dysfunction, fractures, and cognitive impairment^2–5^.

Additionally, the association between Hcy levels and risk of all-cause mortality in human subjects has been explored, but the results are conflicting. In several follow-up studies, Hcy levels were reported to be associated with all-cause mortality^6–8^. For example, Vollset SE *et al*. reported that a 5 µmol/L increment of Hcy was correlated with an increase in all-cause mortality of 49%^9^. However, during an 11-year follow-up, Swart KM *et al*. found that elevated plasma Hcy levels were not related to the risk of mortality in older men^10^. Recently, a meta-analysis^8^ with six studies showed that elevated Hcy levels increased the risk of all-cause mortality in women (RR 1.74; 95% CI 1.24–2.44) and in the whole population (RR 1.93; 95% CI 1.54–2.43), but not in men (RR 1.87; 95% CI 0.64–5.50). Moreover, this meta-analysis with three studies found that the pooled RR of all-cause mortality was 1.27 for a 5-μmol/L increment in serum Hcy in the whole population, implying a possible potential dose-response. However, the small number of studies limits the statistical power and a further dose-response meta-analysis. Therefore, considering the above mentioned findings, it is necessary to assess the association between Hcy levels and risk of all-cause mortality with a larger dataset using a dose-response meta-analysis.

## Results

### Study characteristics

**Figure 1:**
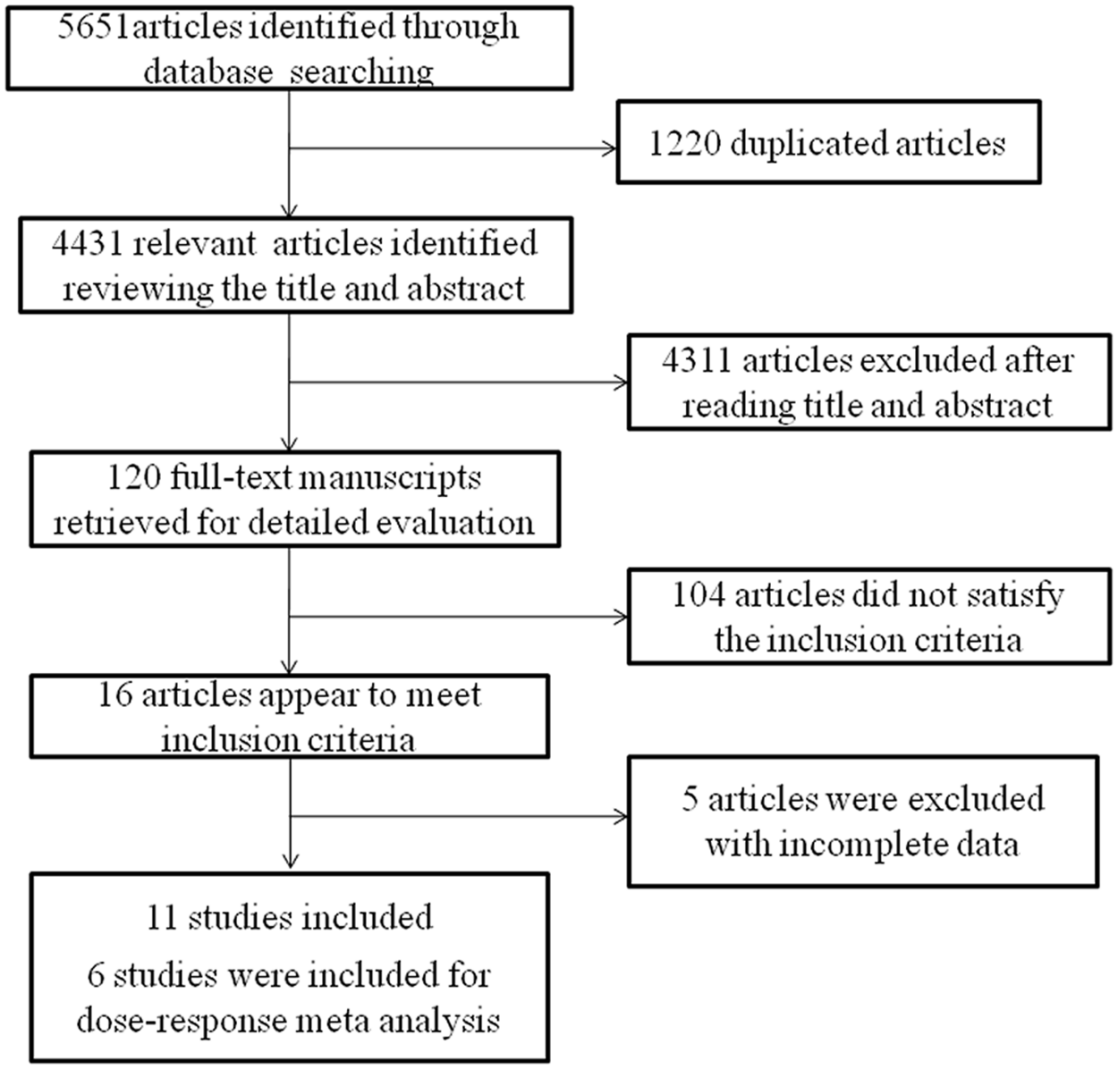
Flow chart of study selection.

The study selection process is presented in Fig. 1. Initially, 5,651 potential studies were identified from the PubMed, Embase, Web of Science, and Cochrane Library databases; however, most of them were excluded according to the exclusion criterion. Finally, 11 eligible studies^7, 9–18^ with a total of 27,737 participants (4,110 deaths) were included. Of the included studies, 3 studies were performed in Asia, 2 in North America, and 6 in Europe. Sample sizes ranged from 215 to 7166, and the median follow-up time varied from 3.08 years to 11 years. Table 1 summarizes the general characteristics of the included studies.

**Table 1:** General characteristics of the included studies.

Study	Year	Study location	Design	duration of follow-up(years)	Male/Female	Age/range Mean(SD)	Study size (Cases/Participants)	Hcy comparison (µmol/L)	Adjustment for covariates	NOS score
Kark, J. D.^16^	1999	Israel	cohort	9–11	808/980	50–92	405/1788	Highest quintile vs.lowest quintile (≥14.7 vs. ≤8.52)	Age, SBP, serum glucose, health status, and serum creatinine concentration	8
Bostom, A. G.^12^	1999	USA	cohort	10	795/1138	70 ± 7	653/1933	≥ 14.26 vs. <14.26	Age, sex, SBP, diabetes,smoking,total and high-density lipoprotein cholesterol levels	7
Hoogeveen, E. K.^15^	2000	Netherlands	prospective nested case-control study	5	case: 100/71control: 297/343	50–75	171/811	>14 vs. ≤14	Age, sex, diabetes, Hypertension, Current smoking,Hypercholesterolemia, Serum albumin,HbA1c	7
Vollset, S. E.^9^	2001	Norway	cohort	4.1	2127/2639	65–67	259/4766	Highest quintile vs.lowest quintile (≥20 vs. ≤8.9)	Total cholesterol, systolic and diastolic blood pressure, pack-years of smoking, BMI, physical activity, age, and sex, cardiovascular disease risk status at baseline	7
Acevedo, M.^11^	2003	USA	cohort	3.08 ± 1.75	2273/1154	56 ± 12	119/3427	Highest quartile vs.lowest quartile (≥14.4 vs. ≤9.4)	Age, Sex, LDL cholesterol, HDL cholesterol, Diabetes mellitus, Hypertension, Smoking, Coronary artery disease	6
González, S.^14^	2007	Spain	cohort	4.3	88/127	75.1 ± 6.5	60/215	Highest quintile vs.lowest quintile (>16.7 vs. ≤8.7)	Age, sex, smoking habit, BMI and cognitive score	7
Dangour, A. D.^13^	2008	United Kingdom	cohort study	7.64	372/481	78.6 (76.8, 81.2)	429/853	Highest tertile vs.lowest tertile (>19.4 vs. ≤9.8)	Age,sex,diabetes,history of CVD,cancer,smoking, alcohol, physical activity,folate, vitamin B-12	8
Xiu, L. L.^18^	2012	China	cohort	10	751/661	65–97	483/1412	Highest quartile vs.lowest quartile (>14.5 vs. ≤9.3)	Age (y), sex, smoking status, BMI, physical function and general health	8
Waśkiewicz, A.^32^	2012	Poland	cohort	5.4	NA	20–74	270/7166	Highest tertile vs.lowest tertile (>10.50 vs. <8.20)	Sex, age, smoking status, hypertension, body mass index and the concentrations of total cholesterol, glucose and high sensitivity-C-reactive protein	6
Wong, Y. Y.^17^	2012	Australia	cohort	5.1 ± 1.3	4248/0	70–88	748/4249	≥15 vs. <15	Age, education, living circumstance, smoking, cardiovascular disease, diabetes, hypertension, dyslipidemia, Charlson comorbidity index, renal function (eGFR), and frailty status at baseline	6
Swart, K. M.^10^	2012	Netherland	cohort	11	543/574	75.1 ± 6.4	513/1117	Highest quartile vs.lowest quartile(M: ≥ 17.57 vs. ≤11.96;F: ≥15.64 vs. ≤10.35)	Age, education level and region,creatinine, body mass index, smoking, alcohol use and physical activity level;, serum vitamin B12	7

### Categorical meta-analysis

**Figure 2:**
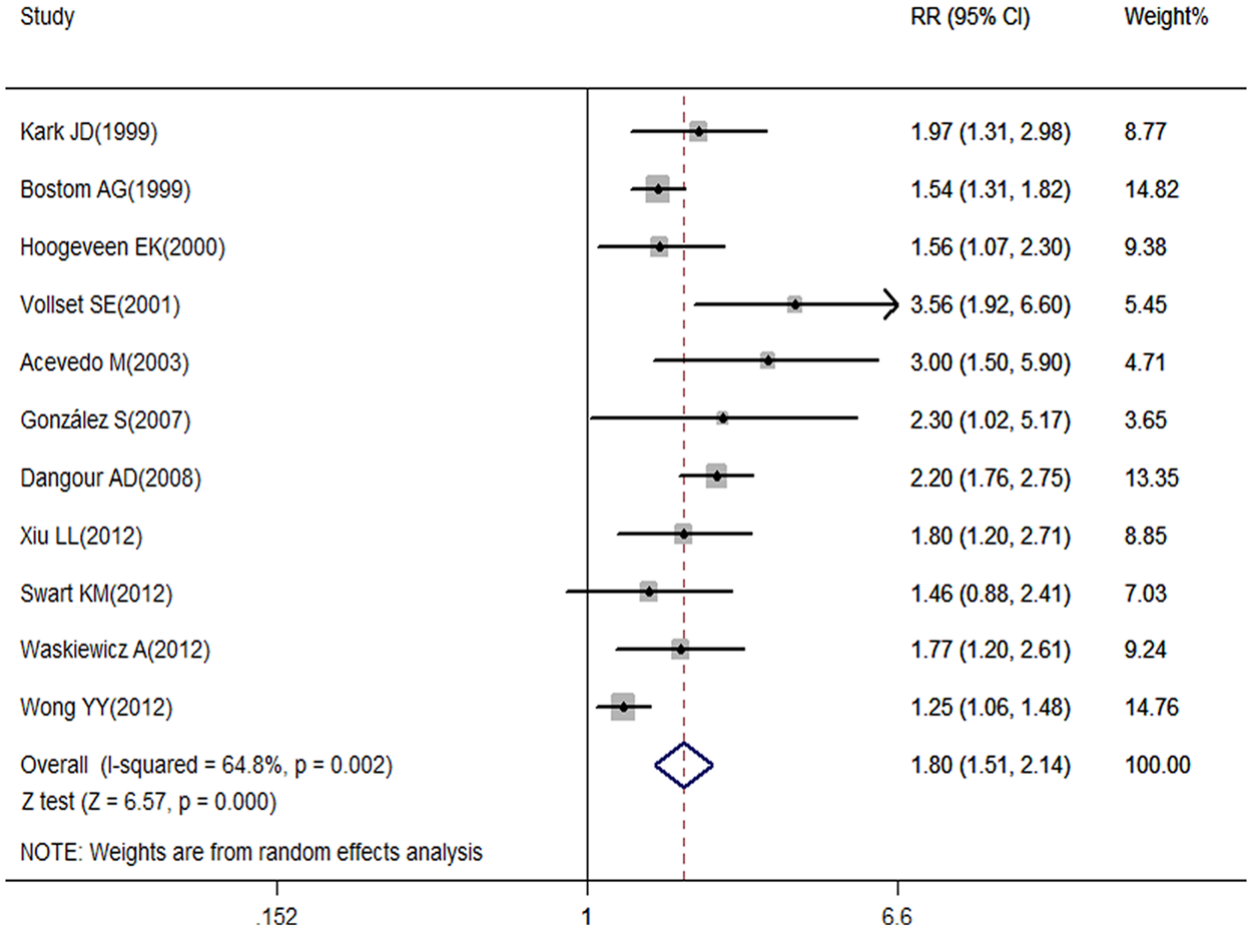
Association between Hcy levels and all-cause mortality risk analyzed by forest plot.

**Figure 3:**
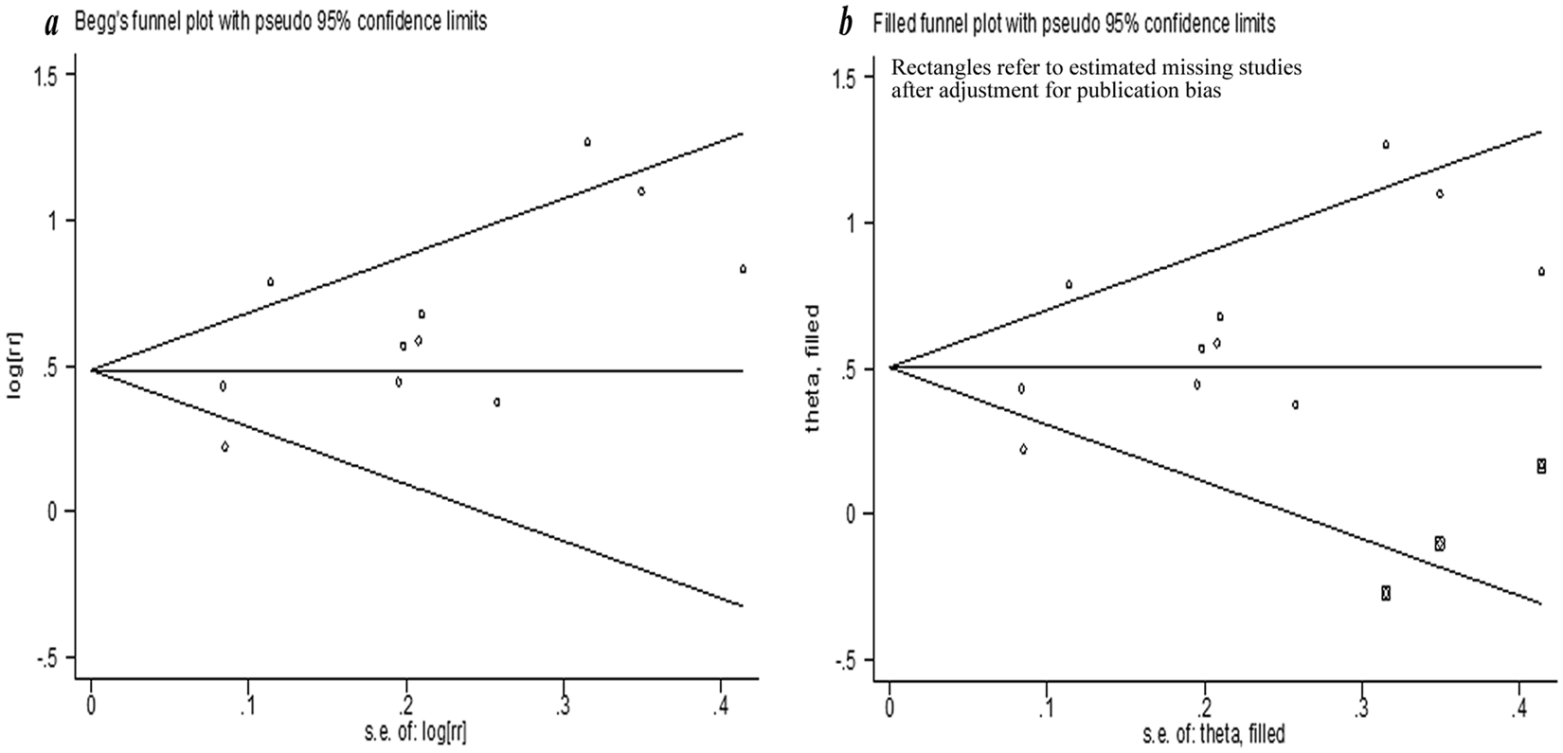
The funnel plot of result.

Our meta-analysis showed that elevated Hcy levels were associated with all-cause mortality risk. Further, the pooled relative risk (RR) (95% confidence interval [CI]) was 1.80 (1.51, 2.14) under the random-effects model with substantial heterogeneity among studies (*I* ^*2*^ = 64.8%, *p*_*H*_ = 0.002, *p*_*z*_ < 0.001) (Fig. 2). According to the funnel plot (Fig. 3a) and Egger’s test (*p* = 0.051), publication bias might exist, although the Begg’s test was not statistically significant (*p* = 0.119). Because of this, we conducted a trim and fill analysis that conservatively imputes hypothetical negative unpublished studies to mirror the positive studies that cause funnel plot asymmetry. The imputed studies showed a symmetrical funnel plot (Fig. 3b) and the pooled analysis continued to reveal a statistically significant association between Hcy levels and all-cause mortality risk (RR = 1.66, 95% CI: 1.39–1.97, *p* < 0.001).

### Dose-response meta-analysis

**Figure 4:**
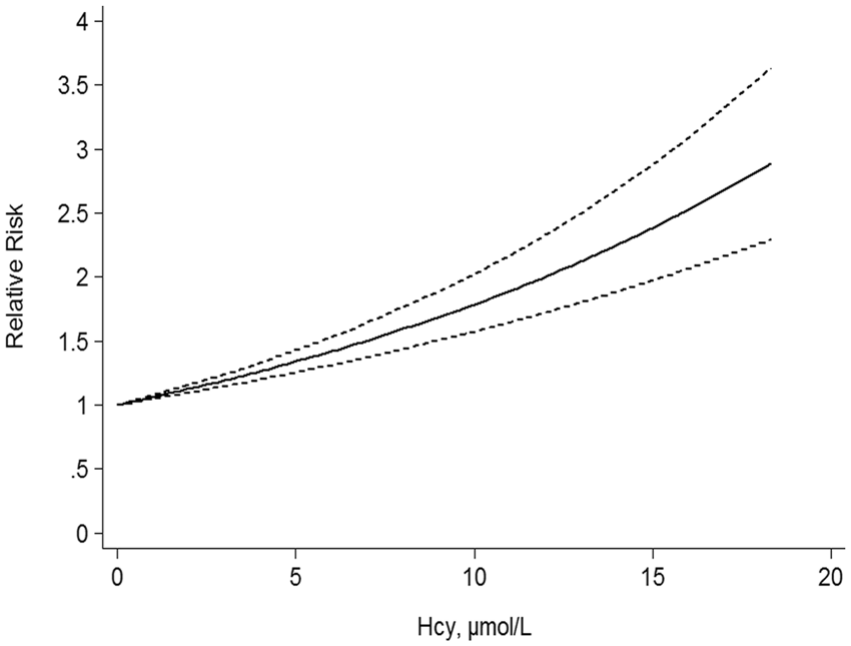
Dose-response relationships between Hcy levels and all-cause mortality risk.

Six studies^7, 9, 11, 13, 14, 16^ were included in the dose-response meta-analysis. The result showed a linear relationship between Hcy levels and all-cause mortality (*p*_non-linearity_ = 0.255), by using a restricted cubic spline model. Furthermore, the linear model revealed an increased all-cause mortality risk of 33.6% for each 5 µmol/L increase in Hcy levels (RR = 1.336, 95% CI: 1.254–1.422, *p* < 0.001) (Fig. 4).

### Subgroup and sensitivity analyses

In subgroup analyses of the association between Hcy levels and all-cause mortality, all subgroups revealed significant positive associations, except for the studies^10, 16, 17^ performed in men (RR = 1.44, 95% CI: 0.96–2.14, *p* = 0.077; Table 2). For studies that adjusted for smoking and diabetes, the pooled RRs (95% CIs) were 1.73 (1.44–2.08) and 1.69 (1.32–2.17), respectively, which were weaker than for those that did not adjust for these factors **(**RR = 2.2, 95% CI: 1.55–3.13; RR = 1.92, 95% CI: 1.58–2.33; respectively). For studies that adjusted for alcohol consumption, body mass index (BMI), physical activity, cardiovascular disease (CVD), and serum vitamin B12/ folate, the pooled RRs (95% CIs) were 1.91 (1.30–2.80), 1.91 (1.53–2.38), 2.10 (1.76–2.50), 1.94 (1.42–2.65), and 1.91 (1.30–2.80), respectively, which were stronger than for those that did not adjust for these factors (RR = 1.76, 95% CI: 1.46–2.12; RR = 1.72, 95% CI: 1.38–2.15; RR = 1.62, 95% CI: 1.35–1.93; RR = 1.62, 95% CI: 1.42–1.86**;** RR = 1.76, 95% CI: 1.46–2.12; respectively).

**Table 2:** The total and subgroup analyses for the relationship between Hcy levels and all-cause mortality risk.

	No. of studies	Relative Risk (RR)		Model of meta-analysis	Heterogeneity		*p*_B_/*p*_E_
		RR (95% CI)	*p*_Z_		*I* ^*2*^(%)	*p*_H_	
**All studies**	11	1.80(1.51, 2.14)	*p* < 0.001	Random-effects model	64.8%	*p* = 0.002	0.119/0.051
**Geographical location**							
Asia	3	1.57(1.14, 2.16)	*p* = 0.006	Random-effects model	65.9%	*p* = 0.053	0.117/0.075
North America	2	1.97(1.05, 3.71)	*p* = 0.035,	Random-effects model	71.0%	*p* = 0.063	0.317/NA
Europe	6	1.99(1.71, 2.33)	*p* < 0.001	Fixed-effects model	35.0%	*p* = 0.174	0.573/0.976
**Sex**							
Male	3	1.44(0.96, 2.14)	*p* = 0.077	Random-effects model	68.0%	*p* = 0.044	1.000/0.502
Female	2	1.74(1.24, 2.44)	*p* = 0.001	Fixed-effects model	0%	*p* = 0.528	1.000/NA
**Duration of follow-up**, **years**							
<7	6	1.91(1.37, 2.67)	*p* < 0.001	Random-effects model	71.9%	*p* = 0.003	0.091/0.008
≥7	5	1.75(1.56, 1.97)	*p* < 0.001	Fixed-effects model	44.5%	*p* = 0.125	1.000/0.760
**Sample size**							
<1000	3	2.03(1.68, 2.45)	*p* < 0.001	Fixed-effects model	16.9%	*p* = 0.300	0.602/0.807
≥ 1000	8	1.74(1.42, 2.12)	*p* < 0.001	Random-effects model	63.5%	*p* = 0.008	0.048/0.017
**Fasting status**							
Yes	4	1.84(1.44, 2.34)	*p* < 0.001	Fixed-effects model	0.1%	*p* = 0.391	0.174/0.145
No	3	1.85(1.43, 2.40)	*p* < 0.001	Random-effects model	70.0%	*p* = 0.036	0.602/0.619
NA	4	1.73(1.17, 2.56)	*p* = 0.006	Random-effects model	75.1%	*p* = 0.007	0.174/0.146
**NOS score**							
<7	3	1.70(1.10, 2.62)	*p* = 0.016	Random-effects model	74.5%	*p* = 0.020	0.117/0.027
≥7	8	1.78(1.60, 1.99)	*p* < 0.001	Fixed -effects model	46.0%	*p* = 0.073	0.216/0.304
**Adjustment for:**							
**Smoking**							
Yes	9	1.73(1.44, 2.08)	*p* < 0.001	Random-effects model	67.2%	*p* = 0.002	0.144/0.154
No	2	2.20(1.55, 3.13)	*p* < 0.001	Fixed -effects model	6.1%	*p* = 0.302	0.317/NA
**Alcohol consumption**							
Yes	2	1.91(1.30, 2.80)	*p* = *0*.*001*	Random-effects model	53.4%	*p* = *0*.*143*	0.317/NA
No	9	1.76(1.46, 2.12)	*p* < 0.001	Random-effects model	60.2%	*p* = *0*.*01*	0.007/0.005
**BMI**							
Yes	5	1.91(1.53, 2.38)	*p* < 0.001	Fixed -effects model	26.7%	*p* = *0*.*244*	0.327/0.334
No	6	1.72(1.38, 2.15)	*p* < *0*.*001*	Random-effects model	75.7%	*p* = *0*.*001*	0.188/0.227
**Physical activity**							
Yes	4	2.10(1.76, 2.50)	*p* < *0*.*001*	Fixed -effects model	45.9%	*p* = *0*.*136*	1.00/0.973
No	7	1.62(1.35, 1.93)	*p* < *0*.*001*	Random-effects model	50.3%	*p* = *0*.*060*	0.051/0.029
**CVD**							
Yes	6	1.94(1.42, 2.65)	*p* < *0*.*001*	Random-effects model	80.8%	*p* < *0*.*001*	0.348/0.140
No	5	1.62(1.42, 1.86)	*p* < *0*.*001*	Fixed -effects model	0%	*p* = 0.667	0.327/0.178
**Diabetes**							
Yes	5	1.69(1.32, 2.17)	*p* < *0*.*001*	Random-effects model	79.4%	*p* = *0*.*001*	0.327/0.340
No	6	1.92(1.58, 2.33)	*p* < *0*.*001*	Fixed -effects model	8.6%	*p* = *0*.*361*	0.188/0.289
**Serum vitamin B12/ folate**							
Yes	2	1.91(1.30, 2.80)	*p* = *0*.*001*	Random-effects model	53.4%	*p* = *0*.*143*	0.317/NA
No	9	1.76(1.46, 2.12)	*p* < *0*.*001*	Random-effects model	60.2%	*p* = *0*.*010*	0.007/0.005

*p*_z_: *p* value of effect test; *p*_*H*_: *p* value of heterogeneity test; *p*_*B*_: *p* value of Bgger’s test; *p*_*E*_: *p* value of Egger’s test. NA: Not applicable.

**Figure 5:**
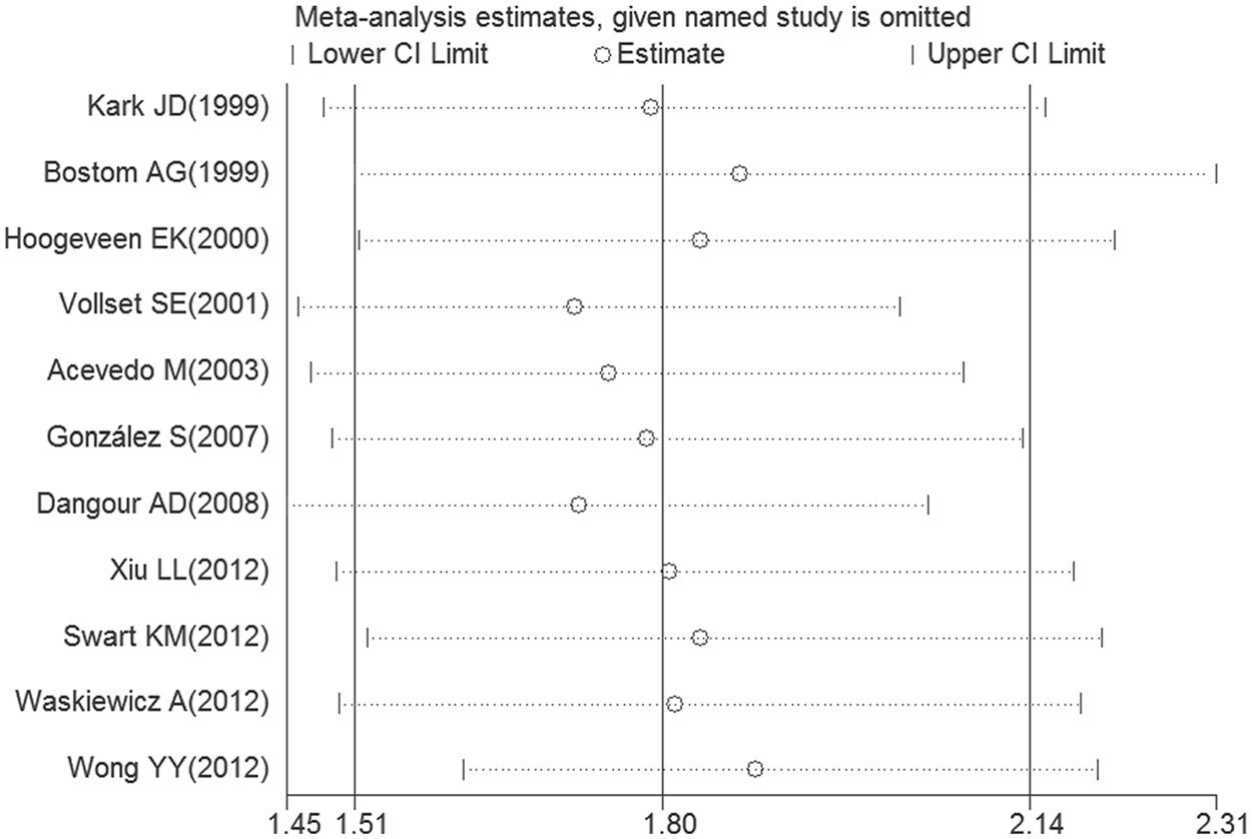
The plot of result of sensitivity analysis.

Sensitivity analysis revealed that no single study significantly changed the pooled results, which indicated that the results of our meta-analysis were robust and reliable (Fig. 5).

## Discussion

It is important to explore whether Hcy levels were correlated with all-cause mortality. In the current meta-analysis, we identified that elevated Hcy levels were associated with the risk of all-cause mortality. Furthermore, the dose-response meta-analysis showed a linear association between Hcy levels and all-cause mortality risk, and each 5 µmol/L increment of Hcy corresponded to a 33.6% increase in risk of all-cause mortality.

Previously, a categorical meta-analysis, by including six studies, found that elevated Hcy levels were associated with all-cause mortality risk, and it found a significant dose-response association between them by including three studies^8^. Although the final conclusion was not significantly changed, there were some differences when comparing the two meta-analyses. First, by searching four databases (PubMed, Embase, Web of Science, and Cochrane Library), the present meta-analysis included more studies (11 studies for categorical meta-analysis and 6 studies for dose-response meta-analysis). Second, although dose response analysis was conducted in both studies, the methods and results were different. In the previous study, the dose response analysis was conducted based on 3 original articles that reported per 5 μmol/L increment in serum Hcy levels. In our study, the restricted cubic spline model was performed to estimate the dose-response relationship. Additionally, we found that Hcy levels were significantly associated with all-cause mortality risk in a linear fashion; this was not reported in the previous study. Third, compared with the previous study, we conducted adequate subgroup analyses, further investigated the potential inter-study heterogeneity, examined the robustness of primary results, and explored the influence of relative factors reported in previous studies.

In addition, Vollset SE *et al*., in a study including 4,766 individuals, reported that a 5-µmol/L increment of Hcy was correlated with a 49% increase in all-cause mortality, and, by using generalized additive logistic regression^9^, the increase in mortality was almost linear. Moreover, using restricted cubic spline, Wong YY *et al*. observed a graded association between Hcy and all-cause mortality^17^. Similarly, with 27,737 individuals included, the present study observed that elevated Hcy levels increased the risk of all-cause mortality in a linear fashion; this further indicated the association between Hcy levels and all-cause mortality. The potential biological mechanisms leading to the positive association between Hcy levels and risk of all-cause mortality are not well elucidated. It is likely that elevated Hcy levels may increase all-cause mortality risk by increasing the risk of major chronic diseases, including cardiovascular disease^19^, fracture^20^, cognitive decline^21^, and endothelial dysfunction^22^. Higher Hcy levels may cause platelet activation and thrombus formation^23^, imbalance between osteoblasts and osteoclasts^24^, hippocampal atrophy^25^, and endothelial dysfunction^22^, leading to cardiovascular disease, cognitive decline, fracture, endothelial dysfunction, which may eventually contribute to mortality. Further studies are needed to indicate the underlying biological mechanisms.

Hcy levels are typically higher in men than in women. Accordingly, several studies revealed that the association between Hcy levels and all-cause mortality might differ by sex. Swart KM *et al*.^10^ reported that elevated Hcy levels increased mortality risk in women only. A previous meta-analysis^8^ included two studies and identified that elevated Hcy levels increased the risk of all-cause mortality in women (RR 1.74; 95% CI 1.24–2.44); however, this positive association was not statistically significant in men (RR 1.87; 95% CI 0.64–5.50). Although we included one more study than the previous meta-analysis, we also did not observe the relationship between Hcy levels and mortality risk in men. Given that most previous studies on higher Hcy levels with mortality risk did not study sex differences, the number of included studies was still limited in the present meta-analysis (only three studies were included), and the results might not be robust enough to draw a conclusion. Thus, it is still unclear whether women or men with elevated Hcy had more mortality risk. Ongoing studies are needed.

Plasma Hcy levels are influenced by life-style and sociodemographic factors. For example, vitamin B and folate, as important cofactors, play an important role in Hcy metabolism. Inadequate dietary supply of vitamin B and folate may lead to hyperhomocysteinaemia^26^. Indeed, numerous studies have indicated that elevated Hcy levels were correlated with low levels of vitamin B and folate^27, 28^. In the present study, we identified that the pooled relative risk for the studies that adjusted for serum vitamin B12/ folate was 1.91 (95% CI: 1.30–2.80); this was stronger than that for studies that did not adjust for this factor (RR = 1.76, 95% CI: 1.46–2.12), suggesting that vitamin B/folate might reduce Hcy levels and thus decrease the risk of all-cause mortality. This subgroup analysis further demonstrated that increased Hcy levels were associated with all-cause mortality.

Although the present study revealed a positive association between Hcy levels and all-cause mortality, the findings should be considered with caution because of the moderate heterogeneity. There are several potential explanations for the observed inter-study heterogeneity. First, with subgroup analyses, we discovered that geographical location, duration of follow-up, sample size, fasting status, and NOS score contributed more or less to the potential heterogeneity, even though no specific factors were found. Second, lack of consistency in the categories of Hcy levels could also have contributed to inter-study differences in the strength of the observed associations. Third, the differences in adjusted confounding factors might have contributed to the heterogeneity to some extent.

A strength of this meta analysis was the prospective design (cohort studies and nested case-control studies were included), which should have greatly reduced the potential of selection bias. Numerous cohort studies with relatively large sample size and long follow-up enabled us to perform informative analysis in various subgroups of populations. In addition, the dose-response meta-analysis further provided a comprehensive description of the association of Hcy levels with all-cause mortality risk. Moreover, in this study, we conducted sensitivity analysis to reflect the influence of each study on the pooled estimates by removing one study at a time. We observed that the estimates did not change significantly, suggesting that the result of this meta-analysis was robust and reliable.

Despite our important findings, there were several limitations to our study. First, selection bias might have been introduced because of our relatively strict inclusion and exclusion criteria and failure to acquire complete data either from the original manuscript or from the authors; however, little evidence for such bias was observed. Second, the subjects in most of the studies were elderly; thus, whether the positive association between Hcy levels and all-cause mortality risk could be generalized to younger persons needs to be confirmed. Third, lack of consistency in the categories of Hcy levels might have contributed to inter-study differences that affected the strength of the identified associations. Fourth, the significant heterogeneity reduced the credibility of our results to an extent that the findings in current studies should be considered with caution. Fifth, publication bias was found according to the funnel plot and Egger’s test, although the trim and fill analysis did not change the general result (though the strength of the association was slightly attenuated), suggesting that the association is not an artefact of unpublished negative studies.

In summary, findings from this dose-response meta-analysis suggest elevated plasma Hcy levels are associated with linearly increasing risk of all-cause mortality. More in-depth studies are warranted to explore the roles of the biological mechanisms and public health interventions aimed at reducing Hcy levels.

## Methods

The current meta-analysis was performed based on the published criteria of the Preferred Reporting Items for Systematic Reviews and Meta-Analyses (PRISMA).

### Search strategy

A computerized literature search was performed in PubMed, Embase, Web of Science, and Cochrane Library (dated up to February 2017) to collect articles regarding the correlation between Hcy levels and all-cause mortality. The following search terms were used: “Hcy” or “homocystine”, or “homocysteine”, or “L-Isomer homocysteine” or “2-amino-4-mercaptobutyric acid”, and “mortality” or “death”. Meanwhile, reference lists of relevant articles were also collected.

### Inclusion and exclusion criteria

The inclusion criteria were as follows: (1) prospective studies including cohort studies and nested case-control studies that explored the association of Hcy levels with all-cause mortality; (2) had reported estimates of RRs with 95% CIs or had presented data with which to calculate these; (3) the publication with longer follow-up and more applicable information was selected when multiple publications on the same study population were available; and (4) articles published in the English language.

The exclusion criteria were as follows: (1) the design was a retrospective study, animal study, meeting abstract, review article, editorial, case report, or other meta-analysis; (2) only unadjusted hazard ratio (HR), RR or odds ratio (OR) were reported, or raw data was unavailable for retrieval; and (3) subjects were in a highly selected disease group.

### Data extraction and quality assessment

Two reviewers (Rui Fan and Aiping Zhang) independently extracted data, cross-checked the data, discussed all conflicts, and reached a consensus on all items. The following characteristics were extracted from each study: first author, year of publication, study location, design of study, duration of follow-up, sample size, age, sex, Hcy comparison, RRs, HRs or ORs with 95% CIs comparing the highest Hcy category with the lowest Hcy category, and adjustment for covariates. For studies that showed several multivariable-adjusted RRs, HRs or ORs, the risk estimates that adjusted for most confounding variables were selected.

The quality of the included studies was assessed independently by the same two reviewers mentioned above, according to the Newcastle–Ottawa Scale (NOS) criteria^29^. NOS scores ranged from 0 to 9, and a score of >7 indicated a high quality.

### Statistical analysis

Categorical and dose-response meta-analyses were performed to assess the association between Hcy levels and all-cause mortality. Pooled RRs with their 95% CIs were used to evaluate the effect size for the association of Hcy levels with all-cause mortality. For studies that showed results separately for females or males, we pooled the results to obtain an overall estimate before combining the results with other studies.

The *I* ^*2*^ statistic was applied to quantify the inter-study differences; an *I* ^*2*^ value of 0% denoted no heterogeneity, 25% denoted low heterogeneity, 50% denoted moderate heterogeneity, and 75% denoted high heterogeneity^30^. The Q statistic was used to formally test for heterogeneity (*p* < 0.10 was considered statistically significant heterogeneity). The fixed-effects model was employed to calculate the RR when heterogeneity was found; otherwise, the random-effects model was applied. In this meta-analysis, subgroup analyses were further conducted to investigate the potential inter-study heterogeneity and examine the robustness of primary results^31^. We divided the subgroups as follows: geographical location (Asia^16–18^, North America^11, 12^, or Europe^9, 10, 13–15, 32^), sex (male^10, 16, 17^ or female^10, 16^), duration of follow-up (<7 years^7, 9, 11, 14, 15, 17^ or ≥7 years^10, 12, 13, 16, 18^), sample size (<1000^13–15^ or ≥1000^7, 9–12, 16–18^), fasting status (yes^7, 11, 14, 15^ or no^12, 13, 16^), and NOS score (≤7^7, 11, 17^ or >7^9, 10, 12–16, 18^). We also performed analyses stratified by whether the studies had adjusted for potential confounding factors, including smoking, alcohol consumption, BMI, physical activity, history of CVD, history of diabetes, and serum vitamin B12/folate.

For dose-response meta-analysis, the included studies should have reported the numbers of deaths and persons/person-years for at least 3 Hcy level categories and the mean or median values of the categories, or have presented the estimated midpoints of the categories if these were not shown in the studies. We assumed the open-ended categories were of the same amplitude as the adjacent categories when the highest categories were open-ended^33^. A 2-stage random-effects dose response meta-analysis was performed to examine the potential trend between Hcy levels and all-cause mortality^34, 35^. This was applied by modelling Hcy levels using a restricted cubic spline model with 4 knots (2 spline transformations) chosen at the 5th, 35th, 65th, and 95th percentiles of the exposure distribution^36^. In the first stage, by taking into account the correlation within each set of specific relative risks, a restricted cubic spline model was fitted to estimate the 2 study-level coefficients and the within-study covariance matrix^34, 35^. In the second stage, by pooling the study-specific coefficient estimates and variance/covariance matrices that had been obtained in the first stage, we derived the overall estimates with random effects^37^. To test the hypothesis that the coefficient associated with the second spline was different from 0, a *p* value for nonlinearity was calculated^35^.

We performed sensitivity analysis by removing one study at a time to calculate the overall homogeneity. Begg’s test,^38^ Egger’s test,^39^ and funnel plot were conducted to assess the presence of publication bias (*p* < 0.10 was considered statistically significant). The trim and fill analysis was also conducted to further evaluate the possible effect of publication bias in our meta-analysis^40^. This method considers the possibility of hypothetical “missing” studies that might exist, imputes their RRs, and recalculates a pooled RR that incorporates the hypothetical missing studies as though they actually existed. All analyses were performed using the software STATA version 12.0 (StataCorp LP, College Station, Texas, USA). Values of *p* < 0.05 were considered statistically significant.
